# Supplementary material for: Rapid and Cost-Efficient Detection of RET Rearrangements in a Large Consecutive Series of Lung Carcinomas
Source: Int J Mol Sci. 2023 Jun 23;24(13):10530. doi: 10.3390/ijms241310530 (PMC10342123; doi:10.3390/ijms241310530)
Supplement: Supplementary file 1 [file ijms-24-10530-s001.zip › ijms-2393323-supplementary.pdf]

Table S1. Frequency of *RET* rearrangements in tumors with various deltaCt values produced by the 5’/3’-end expression test

	dCt >/= 3	3 > dCt >/= 2	2 > dCt >/= 1	1 > dCt >/= 0	0 > dCt >/= -1	-1 > dCt >/= -2	-2 > dCt >/= -3	-3 < dCt	Total
Total	184	385	1587	3966	4510	2797	809	211	14449
Tumors tested for 8 common translocations	169	55	152	1006	1830	1182	367	88	4849
RET-rearranged tumors (8 common variants)	152	18	6	3	5	1	0	0	185
RET-rearranged tumors (common and rare variants)*	163*	19*	6	3	5	1	0	0	197

*Comprehensive analysis for all variants of *RET* rearrangements, which involved variant-specific PCR for common and rare fusions coupled with RNA-based NGS, was applied only to NSCLCs with deltaCt >/=3. Four tumors with deltaCt slightly below 3 were tested by PCR both for common and rare rearrangements. The remaining NSCLCs were analyzed only for eight common translocation variants.

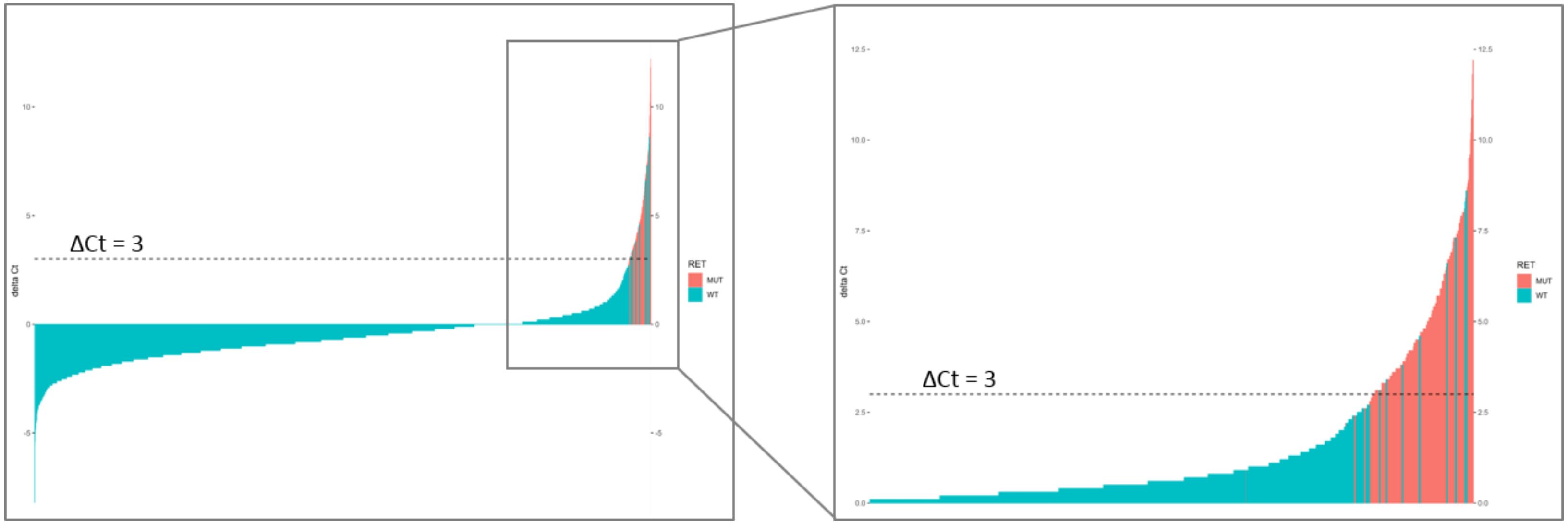

Figure S1. Distribution of *RET*-rearranged and *RET*-wild-type tumors depending on 5’/3’-end expression ratio (dCt).

Table S2. Distribution of *ALK*, *ROS1* and *RET* fusion-positive cases in male and female patients

	NSCLC cases with *RET* fusions	NSCLC cases with *ALK* fusions	NSCLC cases with *ROS1* fusions	Consecutive* NSCLC cases
Total	197	728	204	6153
Female	135 (68.5%)	480 (65.9%)	148 (72.5%)	2005 (32.6%)
Male	62 (31.5%)	248 (34.1%)	56 (27.5)	4148 (67.4%)

*all NSCLC cases referred to molecular testing in the N.N. Petrov Institute of Oncology during the period from May 2021 to February 2023.

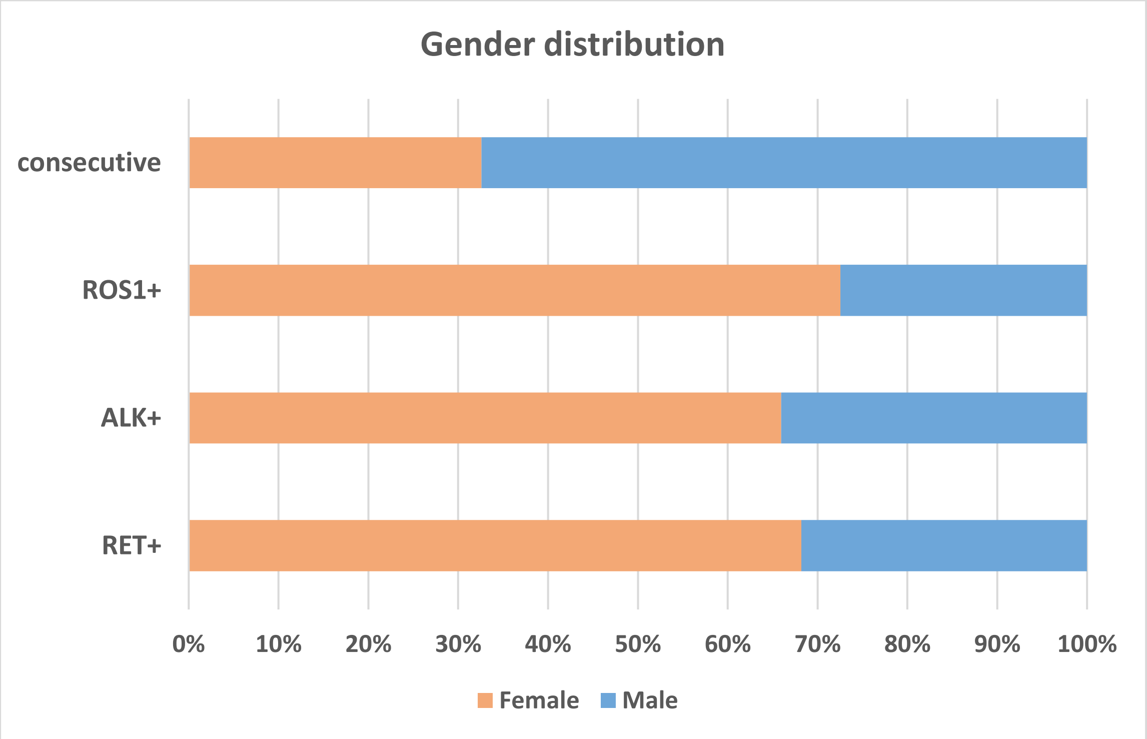

Figure S2. Distribution of *ALK*, *ROS1* and *RET* fusion-positive cases according to gender.

Table S3. Distribution of *ALK*, *ROS1* and *RET* fusion-positive cases according to smoking status and gender

	NSCLC cases with *RET*-fusions (n = 79)	NSCLC cases with *ALK*-fusions (n = 342)	NSCLC cases with *ROS1*-fusions (n = 78)	Consecutive NSCLC cases (n = 2060)*
Smoking status				
Never	61	254	61	993
Female	42 (69%)	198 (88%)	52 (85%)	591 (60%)
Male	19 (31%)	56 (22%)	9 (15%)	402 (40%)
Ever	18	88	17	1067
Female	5 (28%)	17 (19%)	3 (18%)	88 (8%)
Male	13 (72%)	71 (81%)	14 (82%)	979 (92%)

*This Table considers patients with known self-reported smoking status

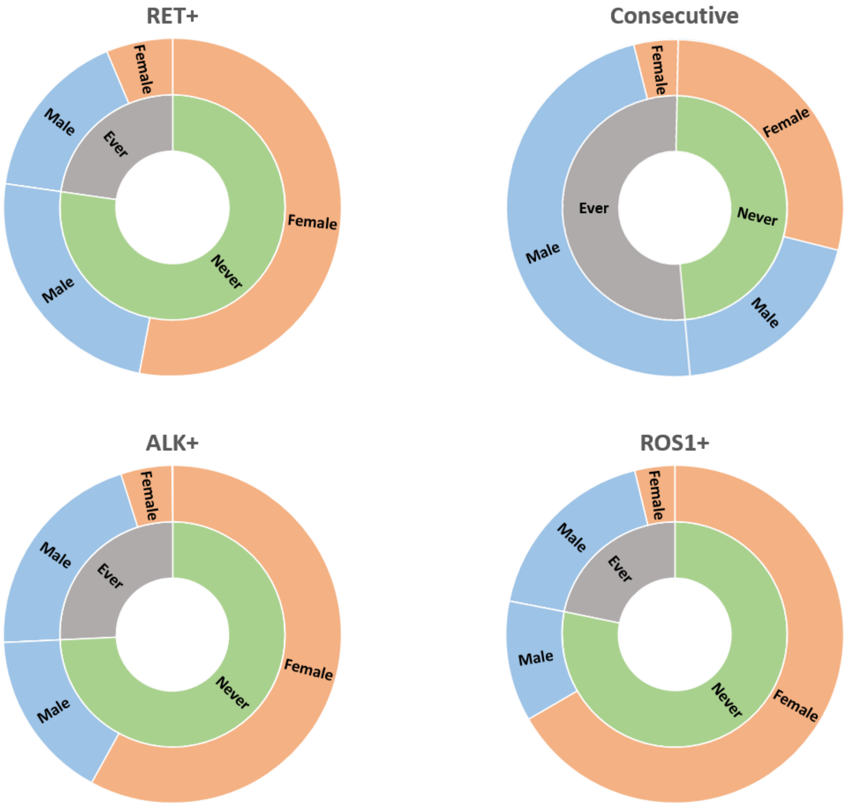

Figure S3. Distribution of *ALK*, *ROS1* and *RET* fusion-positive cases according to smoking status and gender

Table S4. Median age in male and female patients with *ALK*, *ROS1* and *RET* fusions

	NSCLC cases with *RET* fusions		NSCLC cases with *ALK* fusions		NSCLC cases with *ROS1* fusions		Consecutive NSCLC cases	
Median age (age range), years	61 (24-82)		58 (17-89)		59 (22-82)		64 (17-90)	
	Females	Males	Females	Males	Females	Males	Females	Males
Median age, years	63	60	59	58	60	59	64	64

Table S5. Distribution of cases with *ALK*, *ROS1* and *RET* fusions in various age groups according to gender

Age group, years	NSCLC cases with *RET* fusions (n = 197)		NSCLC cases with *ALK* fusions (n = 728)		NSCLC cases with *ROS1* fusions (n = 204)		Consecutive NSCLC cases (n = 6153)	
	Female	Male	Female	Male	Female	Male	Female	Male
	135	62	480	248	148	56	2005	4148
</=30	2 (1%)		17 (2.3%)		4 (2%)		14 (0.2%)	
	2 (1.5%)	0	11 (2.3%)	6 (2.4%)	3 (2%)	1 (1.8%)	7 (0.3%)	7 (0.7%)
31-40	9 (4.6%)		69 (9.5%)		20 (9.8%)		130 (2.1%)	
	5 (3.7%)	4 (6.5%)	40 (8.3%)	29 (11.7%)	16 (10.8%)	4 (7.1%)	71 (3.5%)	59 (1.4%)
41-50	19 (9.6%)		123 (16.9%)		31 (15.2%)		459 (7.5%)	
	11 (8.2%)	8 (12.9%)	78 (16.3%)	45 (18.1%)	19 (12.8%)	12 (21.4%)	174 (8.7%)	285 (6.9%)
51-60	62 (31.5%)		208 (28.6%)		55 (27%)		1466 (23.8%)	
	39 (28.9%)	23 (37.1%)	135 (28.1)	73 (29.4%)	37 (25%)	18 (32.1%)	430 (21.4%)	1036 (25%)
61-70	69 (35.0%)		208 (28.6%)		66 (32.4%)		2819 (45.8%)	
	50 (37.0%)	19 (30.6%)	143 (29.8%)	65 (26.2%)	50 (33.8%)	16 (28.6%)	828 (41.3%)	1991 (48%)
71-80	30 (15.2%)		87 (12%)		26 (12.7%)		1118 (18.2%)	
	22 (16.3%)	8 (12.9%)	63 (13.1%)	24 (9.7%)	22 (14.9%)	4 (7%)	418 (20.8%)	700 (16.9%)
>80	6 (3.1%)		13 (1.8%)		2 (1%)		136 (2.2%)	
	6 (4.4%)	0	9 (1.9%)	4 (1.6%)	1 (0.7%)	1 (1.8%)	75 (3.7%)	61 (1.5%)

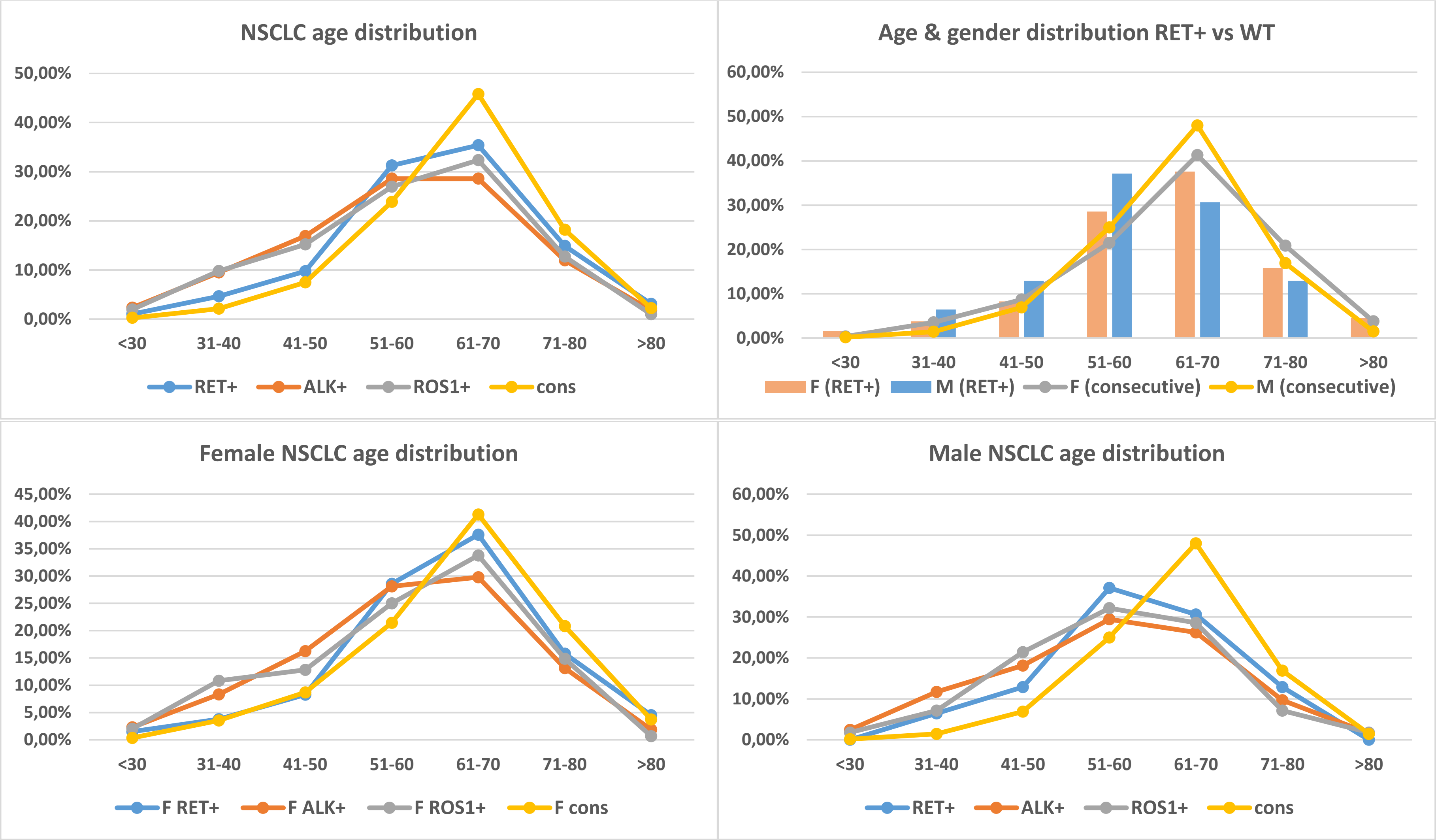

Figure S4. Distribution of cases with *ALK*, *ROS1* and *RET* fusions in various age groups according to gender.

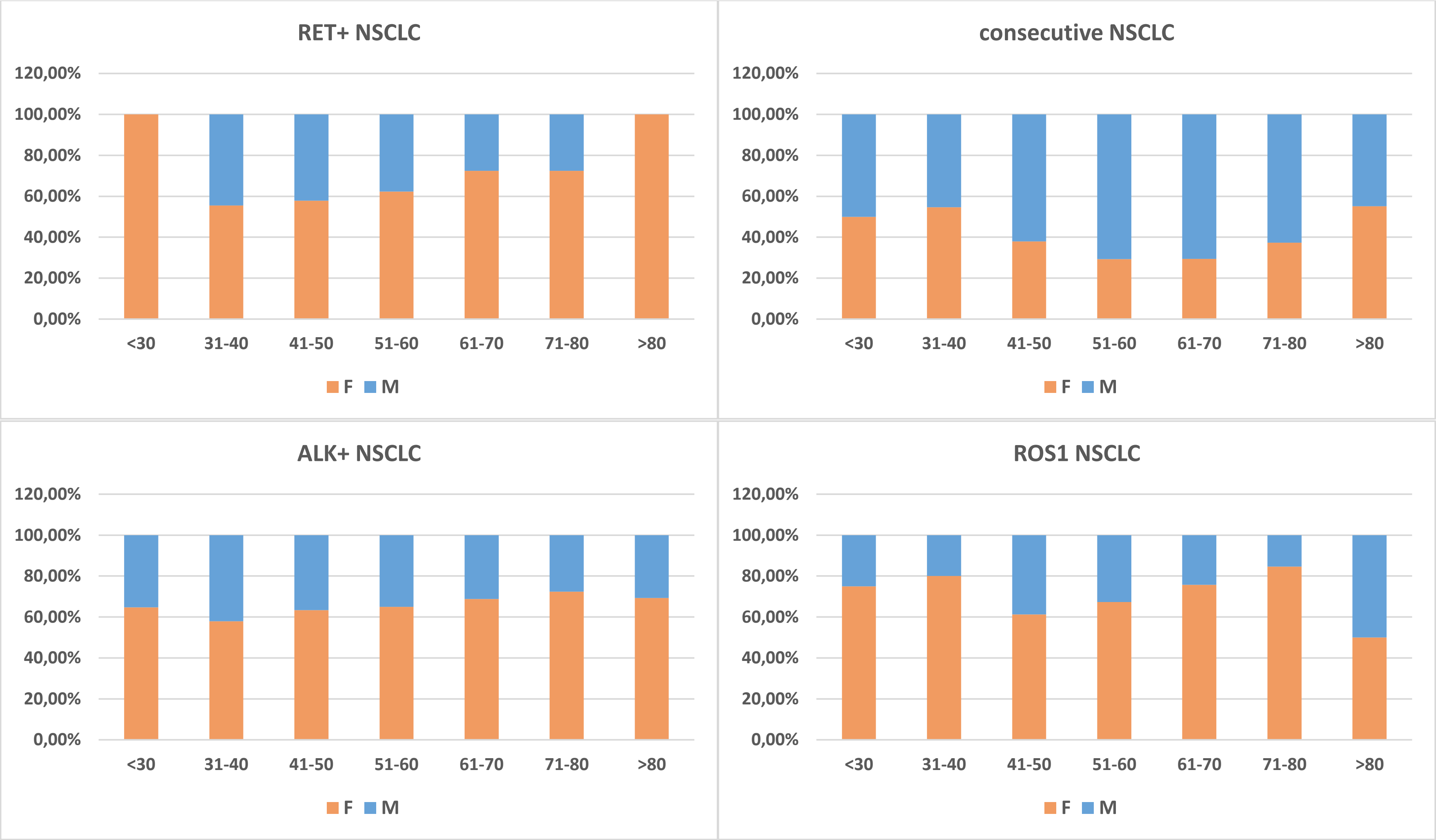

Figure S5. Distribution of cases with *ALK*, *ROS1* and *RET* fusions in various age groups according to gender.

Table S6. Gene-specific primers utilized in RT reaction

Target fragment	Oligonucleotide name	Sequence
SDHA (exons 5-6)	SDHAex6_RT	CACTCCCCGTTCTCCATCA
RET-5' (exons 6-7)	RETex7_RT	CCACTGAATGCCTGGCAGT
RET-3' (exons 13-14)	RETex14_RT	CCCACTTTGCGGCTCTCG
RET variants (exon 12)	RETex12_RT	TCCAAATTCGCCTTCTCCTA

Table S7. PCR primers and probes utilized in the *RET* 5'/3'-end expression imbalance test

Target fragment	Oligonucleotide name	Sequence	PCR fragment length, bp
Test for RNA integrity and amount (amplification of a housekeeping gene fragment)			
SDHA (exons 5-6)	SDHAex5-F	CCACTCGCTATTGCACACC	78
	SDHAex6-R	TCCAAGGCAAATACTCCAC	
	SDHAex5/6-P	R6G-AAGGTCTCTGCGATATGATACC-BHQ2	
Test for *RET* 5'/3'-unbalanced expression (multiplex reaction)			
RET 5'-fragment (exons 6-7)	RETex6-F	AGGAGGGCTCGCCGATTTG	70
	RETex7-R	TGATGCCACTGAATGCCTG	
	RETex6/7-P	FAM-CAGTTTTCCACACAGACTTTCCC-BHQ1	
RET 3'-fragment (exons 13-14)	RETex13-F	TCATCAAATTGTATGGGGCCT	67
	RETex14-R	TATTTGGCGTACTCCACGATG	
	RETex13/14-P	R6G-AGCCAGGATGGCCCGCTCCTC-BHQ2	

Table S8. PCR primers and probes for the variant-specific detection of *RET* translocations*

Fusion	Breakpoint	Oligos for 5'-companions	Oligos for 3'-companion
		For common variants of RET-translocation (multiplex)	
CCDC6::RET	*(C1;R12)*	Forward: GCAAAGCCAGCGTGACCAT	Reverse: TAGAGTTTTTCCAAGAACCAAG<br>Probe: FAM-CTTCCGAGGGAATTCCCACTTTG-BHQ1
KIF5B::RET	*(K15;R12)*	Forward: GCTGTGGGAAATAATGATGTAA	
	(K16;R12)	Forward: AGGAGTTAGCAGCATGTCAG	
	(K22;R12)	Forward: CTTTGTTCAGGACCTGGCTA	
	(K23;R12)	Forward: ATAATCTTGAACAGCTCACTAAA	
	(K24;R12)	Forward: GTCAAAGAATATGGCCAGAAG	
NCOA4::RET	*(N7;R12)*	Forward: AGGCTGTATCTCCATGCCA	
	(N8;R12)	Forward: AGCAGACCTTGGAGAACAGT	
		For rare variants of RET-translocation (multiplex 1)	
CUX1::RET	*(C10;R12)*	Forward: AGATCCAGAAGGCACCA	Reverse: TAGAGTTTTTCCAAGAACCAAG<br>Probe: FAM-CTTCCGAGGGAATTCCCACTTTG-BHQ1
FYCO1::RET	*(F8;R12)*	Forward: GCTGAAATCATGGACTACCA	
ITGA8::RET	*(I30;R12)*	Forward: ACCGACAGGGAACAGC	
KIF5B::RET	*(K19;R12)*	Forward: GTAGAAGCAAAAGCAAAACTTAT	
MPRIP::RET	*(M19;R12)*	Forward: CACAGGGCAAGGATGC	
RELCH::RET	*(R10;R12)*	Forward: CATTGTTCCCAATGTGCTATT	
SLC39A8::RET	*(S6;R12)*	Forward: TCCATTTTGATAATGTCAGTGT	
TRIM33::RET	*(T14;R12)*	Forward: CAGGAGGAGTGCTTGCA	
ZBTB41::RET	*(Z9;R12)*	Forward: AGGCAAATCAAGTCTGGAA	
		For rare variants of RET-translocation (multiplex 2)	
ADD3::RET	*(A1;R12)*	Forward: AGGGAGGGGAAACACAA	Reverse: TAGAGTTTTTCCAAGAACCAAG<br>Probe: FAM-CTTCCGAGGGAATTCCCACTTTG-BHQ1
ANKS1B::RET	*(A1;R12)*	Forward: AGCCCTCGCGGATC	
CCDC186::RET	*(C10;R12)*	Forward: TAGAGTTTTTCCAAGAACCAAG	
FRMD4A::RET	*(F12;R12)*	Forward: GTATGACTACCATGATAAAGTGAA	
KIAA1217::RET	*(K11;R12)*	Forward: CTTTCCTCCTGCGTCAAG	
KIF13A::RET	*(K18;R12)*	Forward: AACCTCAGTGCCAATAGGAA	
MYO5C::RET	*(M25;R12)*	Forward: TCACTTCTGATGGCTTGAA	
RASSF4::RET	*(R3;R12)*	Forward: AGGGCAAGAGCTTCCA	
TBC1D32::RET	*(T9;R12)*	Forward: GTTGATACCAAGGCTGTGT	
WAC::RET	*(W3;R12)*	Forward: ACAGAGTTAGAGAGAGGGATGGT	

Fusion	Breakpoint	Oligos for 5'-companions	Oligos for 3'-companion
For rare variants of RET-translocation (multiplex 3)			
CCNYL2::RET	*(C6;R12)*	Forward: CATATTTGATGAACAGTTACACC	Reverse: TAGAGTTTTTCCAAGAACCAAG<br>Probe: FAM-CTTCCGAGGGAATTCCCACTTTG-BHQ1
LSM14A::RET	*(L9;R12)*	Forward: GAGTTTGCGGATTTTGAATATAG	
PCM1::RET	*(P29;R12)*	Forward: GCAATTATGAAAGAAGTCATTCC	
PRKG1::RET	*(P7;R12)*	Forward: AGGAAAAGGAGACTGGTTT	
PTPRK::RET	*(P3;R12)*	Forward: GCAGTGAGCACCTTTTG	
RUFY2::RET	*(R9;R12)*	Forward: TTAATGAAAACACAGCAGCA	
SIRT1::RET	*(S8;R12)*	Forward: GGCAAAGGAGCAGATTAGTA	
SLC25A36::RET	*(S1;R12)*	Forward: CGCTGGTGCATCTGTT	
SORBS1::RET	*(S8;R12)*	Forward: ATCCCAACCTTCCTTCTGA	
TSSK4::RET	*(T1;R12)*	Forward: TTCCTGCCCCGTGAA	

*all fusions are designated using Ensembl canonical transcripts for the involved genes.

Table S9. PCR primers and probes for the analysis of *TYMS* expression

Target fragment	Oligonucleotide name	Sequence	PCR fragment length, bp
Test for RNA integrity and amount (amplification of a housekeeping gene fragment)			
SDHA (exons 5-6)	SDHAex5-F	CCACTCGCTATTGCACACC	78
	SDHAex6-R	TCCAAGGCAAATACTCCAC	
	SDHAex5/6-P	R6G-AAGGTCTCTGCGATATGATACC-BHQ2	
Test for *TYMS* expression			
TYMS (exons 6-7)	TYMSex6-F	ACACACTTTGGGAGATGCAC	85
	TYMSex7-R	TGGGAAAGGTCTGGGTTCTC	
	TYMSex6/7-P	FAM-AGCCACTGAAAATTCAGCTTCAGCG-BHQ1	

All PCR reactions contained 1 µl of cDNA sample, 1× GeneAmp PCR Buffer I (Applied Biosystems, USA), 250 mkM of each dNTP, 200 nM of each primer and probe, 2.5 mM MgCl2, and 1 U of TaqM polymerase (AlkorBio, Russia) in a total volume of 20 µl. PCR was initiated by the enzyme activation (95°C, 10 min.) and included 38 cycles (95°C for 15 sec. followed by 58°C for 1 min.).
